# HBV Immune-Therapy: From Molecular Mechanisms to Clinical Applications

**DOI:** 10.3390/ijms20112754

**Published:** 2019-06-05

**Authors:** Carolina Boni, Valeria Barili, Greta Acerbi, Marzia Rossi, Andrea Vecchi, Diletta Laccabue, Amalia Penna, Gabriele Missale, Carlo Ferrari, Paola Fisicaro

**Affiliations:** 1 Laboratory of Viral Immunopathology, Unit of Infectious Diseases and Hepatology, Azienda-Ospedaliero-Universitaria di Parma, Parma 43126, Italy; cboni@ao.pr.it (C.B.); barili.valeria@gmail.com (V.B.); greta.acerbi@gmail.com (G.A.); rossi.marzia@gmail.com (M.R.); avecchi2@ao.pr.it (A.V.); dilettal@hotmail.com (D.L.); apenna@ao.pr.it (A.P.); gmissale@ao.pr.it (G.M.); cferrari00@gmail.com (C.F.); 2 Department of Medicine and Surgery, University of Parma, Parma 43126, Italy

**Keywords:** Chronic HBV infection, T cell exhaustion, immune-therapy

## Abstract

Chronic hepatitis B virus (HBV) infection represents a worldwide public health concern with approximately 250 million people chronically infected and at risk of developing liver cirrhosis and hepatocellular carcinoma. Nucleos(t)ide analogues (NUC) are the most widely used therapies for HBV infection, but they often require long-lasting administration to avoid the risk of HBV reactivation at withdrawal. Therefore, there is an urgent need to develop novel treatments to shorten the duration of NUC therapy by accelerating virus control, and to complement the effect of available anti-viral therapies. In chronic HBV infection, virus-specific T cells are functionally defective, and this exhaustion state is a key determinant of virus persistence. Reconstitution of an efficient anti-viral T cell response may thus represent a rational strategy to treat chronic HBV patients. In this perspective, the enhancement of adaptive immune responses by a checkpoint inhibitor blockade, specific T cell vaccines, lymphocyte metabolism targeting, and autologous T cell engineering, including chimeric antigen receptor (CAR) and TCR-redirected T cells, constitutes a promising immune modulatory approach for a therapeutic restoration of protective immunity. The advances of the emerging immune-based therapies in the setting of the HBV research field will be outlined.

## 1. Background

Hepatitis B virus (HBV) is a DNA virus belonging to the Hepadnaviridae family, which includes hepatotropic viruses. The HBV virion consists of an external lipoprotein envelope and an internal protein nucleocapsid with icosahedral symmetry, containing the viral genome and the DNA polymerase. The HBV genome is a partially double-stranded circular DNA molecule with four partially overlapping open reading frames encoding structural and non-structural viral proteins: the core antigen (HBcAg), representing the structural component of the viral capsid; the e antigen (HBeAg), a non-structural protein that is secreted into the serum of the infected host; the large, medium, and small envelope glycoproteins containing PreS1, PreS2 and HBs antigenic reactivities; the DNA polymerase with reverse transcriptase and ribonuclease functions, and the HBV x antigen (HBx), expressing transcription regulatory properties. Following hepatocyte infection, the nucleocapsid is transported into the nucleus, where the viral DNA is converted into a covalently closed circular DNA (cccDNA) in the form of a mini-chromosome which acts as a template for the synthesis of genomic and subgenomic transcripts. Importantly, cccDNA represents a reservoir for virus persistence into the hepatocyte nucleus [1]. HBV DNA fragments can integrate into the host genome, and this event, although not necessary for virus replication, can promote carcinogenesis [2].

Hepatitis B virus infection has been considered by the World Health Organization (WHO) to be a major public health burden because of the high rate of deaths and clinical sequelae, despite the availability of a prophylactic vaccine.

It is estimated that 250 million people worldwide are chronically infected with the hepatitis B virus and at risk of developing liver cirrhosis and hepatocellular carcinoma [3]. Chronic HBV infection can result in a wide range of clinical conditions, associated with variable degrees of HBV control, ranging from chronic viremic patients carrying huge quantities of antigen in their blood and liver, to immune subjects with occult persistence of trace amounts of virus within the liver and without detectable antigenemia. Specifically, five phases have been identified in its natural history, on the basis of the patients’ serological profile and liver inflammation: (i) HBeAg-positive chronic infection (previously referred to as the “immune tolerance phase”); (ii) HBeAg-positive chronic hepatitis; (iii) HBeAg-negative chronic hepatitis (previously referred collectively to as the “immune activation phase”); (iv) HBeAg-negative chronic infection (previously referred to as “inactive carriers”); and (v) HBsAg-negative “occult HBV infection”, with antibodies to HBcAg (anti-HBc), with or without detectable antibodies to HBsAg (anti-HBs), that in case of immunosuppression can lead to HBV reactivation [4].

At present, treatment of chronic HBV infection (CHB) is mainly based on third generation nucleos(t)ide analogue (NUC) therapy, which targets the reverse transcriptase activity of the HBV polymerase, without significant occurrence of viral resistance. NUC are orally administered and well tolerated; they are very effective in suppressing HBV replication, induce biochemical and histological improvement [5,6], and allow a partial restoration of virus-specific T cell responses [7].

Loss of HBsAg is observed in less than 10% of patients after five years of therapy, thus often requiring long-term administration to avoid virus reactivation at therapy discontinuation [5,6]. This is due to the persistence of cccDNA in the nucleus of infected hepatocytes, which is not affected significantly by NUC therapies.

The alternative therapeutic option is based on interferon-alpha (IFN), but an HBV cure is achieved in only 10–20% of IFN-treated patients and therapy is frequently associated with severe side effects [4,8].

Therefore, there is a clinical need for safe, novel treatments to shorten the duration of NUC therapy by accelerating virus control, and to enhance the effect of current anti-viral therapies.

HBV-specific T cells in chronic hepatitis B are scarce and functionally defective and this exhaustion state is a key determinant of virus persistence. Typically, HBV specific T lymphocytes are deeply dysfunctional in untreated chronic patients, while subjects who are able to control HBV infection spontaneously following an acute infection display a vigorous and broad antigen-specific T cell response [9]. Such T cell impairment has been described in animal models of chronic virus infection to be characterized by a progressive and hierarchical loss of antiviral T cell functions, ranging from functional inhibition to physical deletion, depending upon the quantity of antigenic peptides and the duration of T cell exposure to a high antigen load [10].

Further features of exhausted virus-specific CD8 T cells are represented by the up-regulation of multiple co-inhibitory receptors, transcriptional, metabolic and epigenetic defects and the lack of protective T cell memory generation [10].

Additionally, inhibitory mechanisms contributing to T cell dysfunction in chronic hepatitis B comprise nutrient depletion in the hepatic microenvironment, the expansion of negative regulatory T, NK and myeloid-derived suppressor cells (gMDSC), and the effect of suppressive cytokines [11,12,13].

Moreover, different subsets of virus-specific CD8 cells with different levels of exhaustion can co-exist in the same chronically infected host, suggesting that the overall exhausted CD8 T cell population is heterogeneous in terms of phenotypic, functional and transcriptional profiles and capacity to express antiviral function [10].

Based on these lines of evidence, correction of anti-viral T cell defects and boosting virus-specific T-cell responses once T cells have reacquired their capacity to respond efficiently to antigen stimulation represents a rational strategy to cure chronically HBV infected patients. New immunotherapeutic approaches for chronic hepatitis B are currently in clinical development, designed to improve the rate of HBsAg loss and anti-HBs seroconversion in CHB subjects, compared to what is currently achievable with nucleos(t)ide analogues alone.

**Figure 1:**
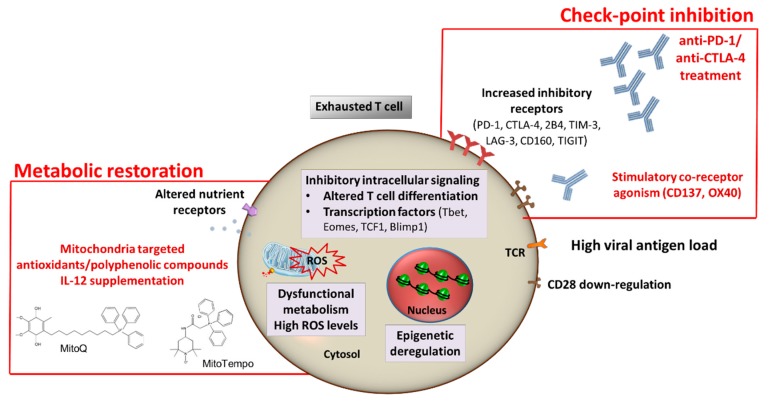
Hepatitis B virus- (HBV)-specific T cell restoration. Different mechanisms can simultaneously operate to inhibit the anti-viral T cell function, including the suppressive effect of the liver environment amplified by inflammation and the high levels of antigenemia. Exhausted T cells are characterized by upregulation of multiple inhibitory receptors (e.g., PD-1, 2B4, LAG-3, CTLA-4, CD160, TIM-3, TIGIT), repressive transcriptional reprogramming (e.g., Tbet and TCF1 downregulation, Eomes and Blimp1 upregulation), broad metabolic alterations (impaired FAO, ROS overproduction and mitochondrial dysfunction), defective T cell effector function (low cytokine production and reduced cytolytic function and proliferation) and memory development. Thus, promising approaches focused on restoring HBV-specific immunity are currently under investigation for chronically HBV infected patients, such as checkpoint blockade and metabolic modulation.

**Figure 2:**
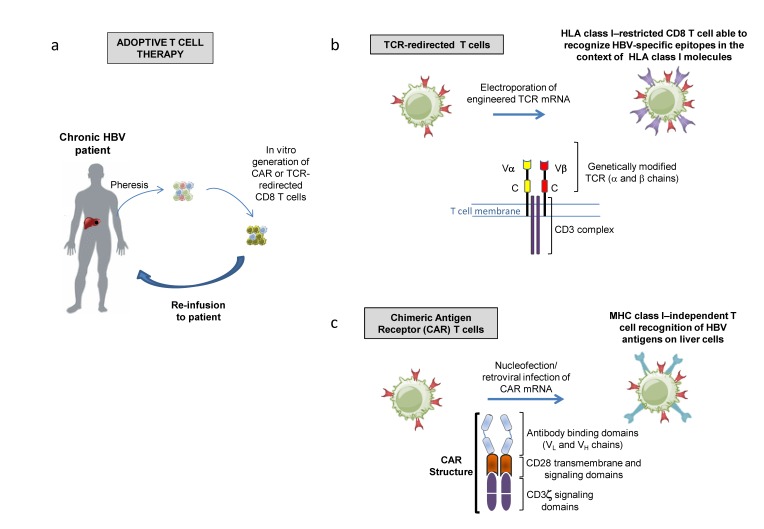
Gene transfer-based strategies as new tools for immune therapy in hepatic tumors and chronic HBV infections. (**a**) Patient-derived CD8 T cells can be modified to express antitumor/antiviral T cell receptors (TCRs) or chimeric antigen receptors (CARs). The infusion of genetically modified T cells targeting specific peptide/HLA complexes or non-processed antigens on the surface of the infected liver cells provides the immune system with functionally efficient CD8 T cells of the desired specificity. (**b**) Schematic representation of an engineered CD8 T-cell with re-directed specificity against a specific HBV epitope. T cells are purified from chronic patients, engineered by electroporation of HBV-specific TCR-expressing vectors and reinfused in the patient. Modified α and β chains of the TCR complex allow high affinity recognition of HLA class I dependent viral or tumor epitopes to reconstitute functional specific immunity. (**c**) CAR T cell engineering is based on the transduction of autologous T cells with an expression vector coding for a chimeric antigen receptor, consisting of an antibody binding fragment (that allows for recognition of conformational antigens, such as HBsAg expressed on the hepatocyte membrane), fused with CD28 transmembrane and CD3zeta intracellular domains, which can mediate constitutive signaling leading to effector T cell activation and HLA class I independent antigen recognition.

This review will focus on the current knowledge about the emerging immune-based therapeutic strategies in chronic hepatitis B virus infection, including immune checkpoint inhibition, metabolic T cell targeted therapies (Figure 1), therapeutic T cell vaccination and autologous T cell engineering, including chimeric antigen receptor (CAR) and TCR-redirected T cells (Figure 2).

## 2. Checkpoint Inhibitors

First reported in chronic Lymphocytic Choriomeningitis Virus (LCMV) infection, the up-regulation of co-inhibitory receptors, or immune checkpoints, has then been widely described as a common hallmark of exhausted CD8 T cells in different chronic infection and tumor models. Indeed, gene expression profiling and functional T cell analysis of virus-specific CD8 T cells from chronically LCMV-infected mice led to the characterization of the role played by PD-1 and other highly co-expressed inhibitory molecules, such as 2B4, CTLA-4, Tim-3, Lag-3, TIGIT, BTLA, CD160, PSGL1, in promoting a dysfunctional phenotype in exhausted antigen-specific CD8 T cells [10]. Later on, the evidence that an efficient antiviral function of exhausted T cells could be reconstituted by blockade of inhibitory pathways promoted expectations on immunotherapy as a potential treatment for chronic infections and cancer. However, while antibodies interfering with PD-1 and its ligands (e.g., anti-PD-1 or PD-L1), both in monotherapy or in combination with other checkpoint targeting, such as CTLA-4, showed some efficacy in cancer treatment [14,15], clinical trials are still very limited in the field of chronic viral infections. In HIV infection, the PD-1 blockade is believed to facilitate virus latency reversal in CD4+ T cells [16]; however, so far only a study has been conducted in ART-suppressed patients with a single low-dose anti-PD-L1 administration leading to HIV-specific T cell improvement in a proportion of subjects, without effect on residual viremia as detected by “single-copy assay (SCA)” [17]. Additionally, in patients with chronic hepatitis C virus (HCV) infection a modest antiviral effect was observed by a single anti-PD-1 dose treatment [18].

In the setting of chronic HBV infection, co-inhibitory molecule expression and its role in T cell exhaustion has been the object of many in vitro and in vivo investigations, documenting also in this context the up-regulation of multiple checkpoints on both circulating and intrahepatic virus-specific CD8 T cells, with maximal expression of PD-1 and 2B4 on liver-infiltrating lymphocytes [19,20,21,22,23,24]. In addition, the overexpression of several checkpoint ligands, such as PD-L1 on circulating monocytes and B cells and on intrahepatic non parenchymal cells, or the TIM-3 ligand galectin-9 on liver resident Kupffer cells [21,25,26], can likely contribute to T cell exhaustion maintenance. Moreover, exhausted HBV-specific T cells also appear more prone to apoptosis, as shown by the up-regulation of the death receptor TRAIL-2 and the pro-apoptotic mediator BIM [27,28,29]. Interestingly, high PD-1 levels have been associated to antiviral dysfunction also in HBV-specific CD4 cells [30], and more recently in virus-specific B cells from chronic HBV patients [31,32]. In addition, recent studies reported that the co-expression of inhibitory checkpoints with several other molecules, such as transcription factors and differentiation/activation/survival markers (e.g., Tbet, Eomes, TCF1, CD127, KLRG1, CD38, Bcl2) can allow to define distinct cell subsets with various degrees of terminal differentiation and functional impairment, with specificity for different viral epitopes, such as HBV core and polymerase [33,34,35,36]. Such phenotypic heterogeneity has already been demonstrated to account for a variable sensitivity to functional restoration interventions in other models of T cell exhaustion [37,38,39,40] and is expected to represent the rationale for the identification of reliable predictors of outcome also in the context of immune therapies for chronic HBV infection. 

Although many in vitro studies showed that the PD-1/PD-L1 blockade can induce some improvement in both the T and B cell arms of the cellular immunity, with a more efficient effect on the intrahepatic than on the peripheral compartment [19,31,32,41,42], it has become evident that the PD-1 blockade alone is not sufficient to completely reverse the immune function impairment in chronic HBV infection [20,21,24,29,43]. Therefore, in order to further improve its efficacy, the PD-1 blockade has been tested in association with other regulatory pathway manipulations, such as TIM-3, CTLA-4, 2B4, CD137, or with IL-12, showing variable degrees of T cell response restoration [44]. Interestingly, a synergistic effect of OX40 (CD134) stimulation with a PD-L1 blockade has been reported to significantly augment IFN-gamma and IL-21 producing HBV-specific CD4 T cells in vitro [45].

Preclinical experiments in the woodchuck model of chronic hepatitis virus (WHV) infection showed that the association of the PD-L1 blockade with nucleoside analogue treatment and therapeutic DNA vaccination boosted virus-specific immunity, leading to suppressed viral replication and anti-WHs antibody seroconversion in two out of three tested animals [46]. A more recent study described an improved control of viremia and antigenemia induced in three of 11 naturally WHV-infected woodchucks when anti-PD-L1 was associated to nucleoside analogue treatment, with durable antiviral effects after therapy withdrawal in two of them [47].

Only a single dose anti-PD-1 (Nivolumab) phase 1 trial has so far been performed in NUC treated, virally suppressed HBeAg negative chronic HBV patients. Reduction of HBsAg titers was detected only in a limited proportion of patients, with a total and persistent HBsAg loss in one of them. No severe adverse events were reported but no further effects were observed on HBsAg decline, and potentiation of antiviral T cell responses by the addition of a therapeutic vaccine containing core envelope and x antigens was not able to stimulate cell-mediated HBV-specific responses [48]. Given the wide variability in the efficacy of checkpoint inhibition in both in vitro and in vivo studies, the complexity of the individual response to the treatment is currently being investigated, with the aim of identifying the fraction of patients who could more likely benefit from immunotherapies. Genetic and epigenetic factors, which can contribute to the variable individual checkpoint expression and to the development of the T cell exhaustion state, have been recognized [49]. In this regard, heritable de novo DNA methylation programs that affect the T cell function and persist upon the PD-1 blockade with possible resistance to immune modulatory treatments and to reversal of exhaustion have been described in mouse models of chronic virus infection [50]. Since exhausted T cells undergo deeply altered transcriptional regulatory programs [51,52], a combination of epigenetic drugs, such as FDA-approved DNA demethylating agents, with an immune checkpoint blockade has already been tested and demonstrated to be effective in improving anti-tumor immunotherapy [50].

Although promising, the clinical use of an immune checkpoint blockade may however be limited by potential side effects [53]. These may be particularly related to a generalized immune activation induced by checkpoint blockade, which could give rise to autoimmune events, as well as to increased liver inflammation, that might lead to several degrees of unwanted consequences, including hepatitis exacerbation, as reported in HBsAg-positive cancer patients [54,55,56]. Moreover, a recent study performed in HBsAg-transgenic (tg) mice upon TIGIT-inhibition showed that therapeutic HBsAg vaccination further induced inflammation and hepatocellular carcinoma (HCC) in a CD8 T cell-dependent manner [57]. These data underscore the risks of interventions affecting hepatic immune tolerance, in view of the evidence that recurrent immune-mediated liver damage can contribute to the development of cirrhosis and HCC [58]. 

## 3. Metabolic Modulation

T cell differentiation, activation and function, as well as memory generation, require dynamic metabolic adaptations in order to cope not only with different biosynthetic and energetic cellular demands, but also with changes in nutrient availability due to T cell migration from one tissue to the other [59]. Thus, it is becoming increasingly clear that the immune cell metabolism ultimately shapes the immune response. Many studies have so far been devoted to understanding the interdependence between T cell metabolism and function in order to identify metabolic modulation strategies relevant to therapeutic applications in different clinical fields where T cell exhaustion is pathogenetically relevant [60]. An example of a mitochondrial manipulation influencing T cell dynamics has been provided by the effect of mitochondrial fusion-promoting drug treatment in improving the cell fitness and function of adoptively transferred anti-tumor CD8 T cells in mice [61]. Moreover, a dysregulated metabolism has been described in the mouse model of LCMV infection where early metabolic alterations precede the onset of severe T cell dysfunction [62], with suppression of both glycolysis and mitochondrial respiration associated with significant transcriptional changes. Virus-specific CD8 T cells containing abnormally large, depolarized mitochondria with a subverted ultrastructure and increased ROS production persisted also in advanced stages of exhaustion and were enriched in the more terminally differentiated PD1hiEomeshi CD8 T cell subset. A PD1 blockade could partially relieve such metabolic alterations, mostly in PD1int cells, in line with previous reports demonstrating the influence of inhibitory receptor signaling on T cell metabolism [63]. Overexpression of the transcriptional co-activator PGC-1α can reverse the dysregulated mitochondrial phenotype and can significantly increase T cell polyfunctionality. The same approach has also been applied to rescue exhausted tumor-infiltrating lymphocytes, with a positive effect of PGC1α over-expression on glucose uptake, glycolysis and mitochondrial dysregulation, as well as on anti-tumor functions [64,65].

Similarly to what is described in tumors, nutrient competition and restriction in the tumor microenvironment as well as the inhibitory effects of accumulated metabolites [66] represent factors responsible for T cell dysfunction also in the infected liver, where amino acid depletion caused by their consumption by a number of infiltrating cells has been described as part of the immunosuppressive environment [11,67]. The decrease in arginine levels by the granulocytic subset of myeloid-derived suppressor cells (gMDSC) producing arginase I, has been shown to be one way of depriving T cells of this essential amino acid, transiently in acute HBV patients and more persistently in chronically infected individuals, particularly in the infection phases without overt immunopathology, such as in immunotolerant and inactive patients [13]. This finding has been further confirmed by partial CD8 cell functional reconstitution following in vitro arginine replenishment [68]. The inflamed hypoxic hepatic environment may also contribute to the significant GLUT-1 up-regulation observed in exhausted HBV-specific CD8 T cells, that have been described as being heavily dependent on glucose up-take and glycolysis for energy production, because of their limited capacity to use oxidative phosphorylation, in comparison with more functional CMV-specific CD8 cells. As in LCMV, also in chronic HBV infection virus-specific CD8 T cells display abnormally large, depolarized, dysfunctional mitochondria, that can be rescued by IL-12 addition to the culture medium [69]. Mitochondrial dysfunction with a high proportion of depolarized mitochondria and excessively high ROS levels has also been supported in chronic hepatitis B by a genome-wide transcriptome study of virus-specific CD8 cells, showing an extensive downregulation of genes coding for different mitochondrial components of the electron transport chain (ETC), fatty acid and amino acid metabolism and heme biosynthesis [70]. Among the reported down-regulated genes in exhausted HBV-specific CD8 cells, those coding for the mitochondrial membrane fusion protein OPA1, the mitochondrial enzyme CPT-1α, and the costimulatory receptor CD28 could represent factors affecting the correct T cell differentiation, in consideration of their demonstrated role in metabolic plasticity and functional memory in T cell development [61,71,72]. 

In this context, mitochondria-targeted antioxidants, by neutralizing the excess ROS production, resulted in high in vitro response rates, as shown by significant improvement of mitochondrial depolarization and ETC protein expression, as well as HBV-specific T cell cytokine production and viability, not only at peripheral but also at intrahepatic level [70]. Considering the multifaceted roles played by mitochondria in a number of cellular processes, all of which can ultimately impact on T-cell proliferation and the effector function, mitochondrial modulation should be considered in the perspective of immune-modulatory strategies targeting multiple dysregulated cellular processes. It can be an alternative or complementary approach to the checkpoint blockade in order to restore T cell function and responsiveness to antigen stimulation and render HBV-specific T cells efficiently responsive to boosting vaccination.

## 4. Therapeutic Vaccination

Since the spontaneous resolution of HBV infection is accompanied by immune reconstitution, stimulation of HBV-specific B and T-cell immunity by therapeutic vaccination in the context of a chronic infection represents a rational approach to overcoming immune tolerance. Therapeutic vaccination is an attractive field of research, nevertheless different therapeutic vaccine attempts in hepatitis B have so far been unsuccessful. To date, several formulations have been tested both in animal models and humans, and different categories of immunogens have been developed, including protein- or peptide-based, DNA- and viral vector-based vaccines. Many different vaccine therapies for chronically HBV infected patients have already been evaluated in clinical trials [73,74,75,76] (Table 1) and some of them are discussed below.

Since adenoviruses have emerged to provide optimal stimulation to the T cell compartment, some strategies have been designed to exploit such properties. Among viral vector-based vaccines, TG1050 consists of a non-replicative adenovirus 5 vector encoding a unique fusion protein composed of modified core, polymerase and selected domains of the envelope proteins. Injection of TG1050 was able to induce a robust T cell response and to exert an antiviral effect in HBV-persistent mice [87]. It is currently under evaluation in a Phase Ib study in chronic patients with inhibition of HBV replication induced by antiviral therapy (NCT02428400). 

To improve therapeutic vaccine efficacy, a novel approach called TherVacB based on a modified vaccinia virus Ankara (MVA) vector expressing HBsAg and HBcAg has been proposed [88,89]. It was shown to elicit strong polyclonal CD4 and CD8 T cell responses inversely correlated with antigen levels, in HBVtg mice [76,89]. This strategy, currently at pre-clinical evaluation, has been designed as a two-step approach, based initially on protein priming of HBV-specific CD4 T cell responses for CD8 T cell help, and antibody production to lower HBsAg titers, followed by subsequent boosting by the MVA vector in order to optimize the efficiency of activation and expansion of the HBV-specific CD8 T cell response.

Among the candidate vaccines belonging to the protein-based category, GS-4774 consists of a heat-inactivated, recombinant Sacchyaromyces cervisiae yeast that expresses HBsAg, Core and x antigens [90]. The yeast component has been shown to have adjuvant properties and to reduce frequency and inhibitory activity of T regulatory cells (Tregs) [91,92]. GS-4774 has been tested in a phase 2 trial in virally suppressed CHB patients and more recently in naïve chronic patients in combination with tenofovir [77,78]. In both studies GS-4774 did not induce clinically significant reductions in HBsAg, although it could efficiently promote virus-specific CD8 T-cell responses breaking T-cell tolerance in viremic HBeAg-negative patients [78]. The absence of clinical benefits, despite the strong CD8-mediated immune modulatory effect, is probably due to the weaker CD4 T cell stimulation, in view of the essential role played by CD4 T cells in inducing B cells and neutralizing antibodies. Moreover, the partial restoration of envelope-specific T cell responses observed in this study could represent another important cause of the vaccine failure in reducing HBsAg load, in line with the concept that envelope-specific responses are associated with complete control of infection and anti-HBs seroconversion [7]. 

Further novel protein-based vaccine candidates incorporating HBsAg and HBcAg and currently in clinical trials are Theravax (DV-601), combined with a saponin-based ISCOMATRIX adjuvant [79], and ABX203, administered by the intranasal route [80]. In addition, an innovative vaccination approach consists of the yeast-derived HBsAg and human anti-HBs immunoglobulin complex combined with alum adjuvant (HBsAg-HBIG) [81,82]. 

In general, these data support the idea that therapeutic vaccination based on a rational choice of antigens and appropriate adjuvants/viral vectors should be able to induce multi-specific and broadly cross-reactive HBV-specific CD4 and CD8 T cell responses associated with a restoration of T cell reactivity to envelope, polymerase and core. The antigen choice represents a crucial point in the immunization strategy, since probably the use of a therapeutic vaccine targeting only HBsAg may not be optimal to achieve clinical and immunological success. Moreover, the role of adjuvant has emerged as a further critical component to overcome different mechanisms of T cell dysfunction, in view also of the recent evidence that vaccine-adjuvant systems may be utilized to induce beneficial epigenetic modifications in the setting of anti-cancer treatment [93].

Among the new vaccines belonging to the peptide-based category at present in clinical trials, HepTcellTM is composed of nine multi-epitope peptides including CD4 and CD8 T cell epitopes targeting multiple HBV genotypes (NCT02496897), and ePA-44 consists of immunodominant epitopes derived from PreS2 18-24 region, core-18-27 and tetanus toxoid (NCT01326546).

DNA-based vaccines have also been studied and some are currently in clinical trials [84,85,86]. One novel candidate is INO-1800, consisting of plasmids encoding HBsAg and a consensus sequence of HBcAg, which is administered with or without INO-9112, a human IL-12 DNA plasmid [94] (NCT02431312). HB-110 is another new generation DNA vaccine containing plasmids encoding HBs, PreS1, HBc, HBpol and IL-12 [83]. Both vaccines need to be administered by in vivo electroporation. Interestingly this approach has been reported to enhance vaccine antigen expression and immunogenicity. Moreover, IL-12 has been used as an adjuvant to rescue the antiviral function of exhausted HBV-specific T cells [43].

In conclusion, some general considerations on therapeutic vaccination efficacy can be outlined. First, an ideal approach should aim at achieving a functional restoration of CD4, CD8 T and B cell responses, likely needed for long-term control of HBV infection. Furthermore, an important open issue is also the optimization of therapeutic vaccination through the addition of other immune-modulators, such as checkpoint inhibitors and metabolic modulators to overcome HBV-specific immune exhaustion. Finally, lowering the antigen load before T cell boosting to minimize the risk related to hepatic flares and to favor functional T cell restoration as well as an accurate selection of patients that could be more likely to respond to immune modulation represent two further crucial aspects to consider in the vaccine design strategy. With respect to the last point, a better characterization of T cell exhaustion heterogeneity should help identify the patient populations with a prevalence of exhausted T cell subsets that are better responsive to T cell revitalizing strategies and that could thus benefit more substantially from immune modulatory interventions.

## 5. Adoptive Transfer of Genetically Engineered T Cells

To what extent T cell exhaustion can be overcome, and whether achievable functional restoration is sufficient for HBV control are still open issues that need to be better addressed. In this setting, T cell engineering technologies aimed at generating in vitro functionally efficient T lymphocytes, with re-directed specificity against HBV, to be re-infused in the chronically infected host represent alternative strategies to overcoming the possible inefficiency of immune modulatory therapies in the stimulation of impaired HBV-specific T cell responses (Figure 2).

The vast majority of genetically engineered T cell adoptive transfer therapies have so far been targeted to treat malignancies, such as lymphoma, leukemia and neuroblastoma [95,96]. Recent reports indicate that adoptive cell transfer currently comprised of TCR-engineered and chimeric Ag receptor (CAR)-T cells have the potential to treat a variety of other diseases including multiple sclerosis, inflammatory intestinal diseases and autoimmune diseases [97,98,99]. Moreover, in the last few years researchers have also considered extending T cell engineering to therapies for virus infections such as HIV-1, CMV, HBV, HCV and SARS [100,101,102,103,104,105,106,107]. In this regard, clinical evidence of the potential effectiveness of immune-modulatory strategies designed to strengthen HBV-specific T-cell responses by adoptive transfer of engineered antigen-specific T lymphocytes are based on the observation that virus-specific T cell administration through a bone marrow transplant from subjects who cleared HBV infection spontaneously, to patients with chronic HBV infection, led to virus control [108]. Similarly, liver transplantation in a patient with resolved HBV infection receiving a HBsAg positive graft resulted in viral clearance [109]. Thus, adoptive T-cell therapy using autologous T cells genetically engineered to express a canonical HLA class I restricted TCR, or a chimeric antigen receptor (CAR), targeting both HBV and HCV chronic viral infections has been attempted [103,104,105,106,107]. In HBV infection, the re-directed specificity of existing T cells by transfer of HBV-TCR genes has so far been tried in HBV transgenic mice and in patients with relapses of HBV-related HCC [110,111]. Briefly, circulating T lymphocytes isolated from CHB patients have been expanded and activated in vitro and then engineered using viral vectors encoding HBV-specific TCR to redirect their specificity towards HBV. The modified and fully functional T cells have then been re-infused into the chronically infected patients and through this technology engineered lymphocytes have been able to recognize and lyse HBV infected hepatocytes. Importantly, HBV-specific TCR reprogrammed T cells were capable not only of causing a drop of HBsAg produced by HCC cells with integrated HBV-DNA in an HBsAg positive patient with hepatocellular carcinoma (HCC) [110], they were also able to induce the reduction of pulmonary metastases in another case of HBV-related HCC, that was negative for HBV antigen expression analysis by immunohistochemistry. In this latter study, short HBV-DNA fragments integrated into the HCC cells’ genome were sequenced and used to select the HBV-specific TCRs for individualized engineered T cell immunotherapy [112]. HBV-specific TCR T cells are HLA-class I restricted; therefore, they can recognize viral peptides only in the context of the appropriate presenting HLA molecule. An important advantage of this approach is that HBV-specific TCR-T cells do not recognize circulating antigens and thus they are not inhibited by the high quantity of soluble antigens typically present in the serum of CHB patients. Moreover, T cells that transiently express HBV-specific TCR have been recently generated through messenger RNA (mRNA) electroporation technology and adoptively transferred in HBV infected human liver chimeric mice [113]. Thanks to their short life span, by this strategy engineered T cells have been adoptively transferred in escalating doses, thus reducing the risk of potential liver toxicity and inducing a progressive immune-mediated viremia reduction. In addition, as a safer approach to avoid the risk of severe liver damage, the same researchers constructed TCR-reprogrammed, non-lytic T cells able to produce low amounts of perforin and granzyme B but capable of limiting viral infection by activation of the anti-viral cytidine deaminases APOBEC3 in HBV-infected hepatocytes [114].

As an alternative strategy, T cells able to recognize HBV-infected targets independently of the patient HLA haplotype have been engineered using a chimeric antigen receptor (CARs) that combines an HBV-specific antibody fragment with the co-stimulatory CD28 molecule and the CD3 zeta intracellular domain. These chimeric receptors have been retrovirally delivered in primary human T cells and by this technology they could recognize conformational, non-processed antigen on the cell surface in an MHC class I-independent manner. In this regard, CAR T cells directed against the HBsAg protein (S-CAR) enable primary human T cells to recognize and kill HBV infected hepatocytes expressing HBsAg on their surface and to eliminate viral cccDNA in vitro [104]. Moreover, in an HBV transgenic mouse model, CD8 T cells expressing the chimeric antigen receptor specific for HBV envelope proteins were localized in the liver after adoptive transfer and were able to reduce HBV replication [111]. However, a very recent study highlighted a limitation of the immunocompetent transgenic mouse model, describing human S-CAR cell rejection by the murine immune system. This hurdle has been recently overcome by specifically inducing tolerance against the human-derived CAR domains, leading to persisting S-CAR cells with antiviral effect [115]. 

A different model for the study of HBsAg-CAR T cells has recently been generated in persistently HBV-infected chimeric immunodeficient mice, harboring a humanized liver with viral cccDNA into infected hepatocytes. HBV-DNA and HBsAg level reduction by transferred CAR T cells has been reported, also in these animals [116].

Based on these observations, adoptive transfer of redirected T cells represents a potential immune-modulatory approach for a therapeutic reconstitution of anti-viral protection. 

In this perspective, there is a specific concern regarding the risk that, once re-administered to the patient, engineered T cells may undergo functional inhibition by the same suppressive mechanisms responsible for T cell exhaustion of endogenous antigen-specific lymphocytes. Indeed, adoptively transferred T cells have been reported to display susceptibility to inhibitory co-receptor-mediated exhaustion [117]. This issue has been addressed in cancer models through the association of checkpoint blockade to CAR T cell therapy or by an engineered down-modulation of checkpoint inhibitor expression on T cells [118]. A recent study describes the shRNA knockdown of PD-1 in TCR-redirected T cells, as well as in intrahepatic lymphocytes from CHB and HCC patients, through a lentiviral vector transduction. These engineered T cells demonstrated enhanced functionality in culture, and also in a microfluidic model of 3D culture recreating some characteristics of the tumor microenvironment, such as high PDL-1 expression. However, upon repetitive antigenic stimulation, PD-1 knock-down led to a compensatory increase of alternative co-inhibitory receptors and to a phenotype with features of apoptosis and senescence, thus demonstrating that engineered T cell therapy still needs to be further refined in order to be highly effective [119].

Among different approaches studied to further improve genetically modified T cell therapy, recent reports have highlighted the importance of modulating in vitro CAR-T cell metabolism before their transfer in vivo. Interestingly, given the growing understanding of metabolic dysregulation as a key alteration in exhausted CD8 T cells, improving adoptive cellular immunotherapy through metabolic preconditioning of CAR-T cells has currently being addressed in the setting of malignancies. Interventions aimed at promoting memory T cell-like metabolism and oxidative phosphorylation (OXPHOS), and at reducing aerobic glycolysis, have been attempted to optimize metabolic profiles of immune T cells in vitro before adoptive transfer [66].

Although a growing number of studies are currently addressing engineered T cell therapy challenges and potentials, at present its clinical management in the setting of chronic HBV infection remains technically complex and difficult to apply for a large number of patients.

## 6. Final Remarks

Restoration of functionally efficient adaptive responses is believed to be a possible strategy to control HBV infection in chronically infected patients, but the proof of principle that this can actually be achieved in vivo is still lacking. There is evidence in different models of chronic virus infection that a persistently elevated antigen load can inhibit T and B cell functions, but no data are available in chronic HBV infection to support the notion that the decline of antigen can allow T and B cell functional reconstitution. Despite this, inhibition of antigen production may actually represent the first possible strategy to be put in place to reconstitute adaptive responses, in consideration of the high antigenemia constantly present in chronically infected patients. To further amplify this effect, checkpoint blockade and metabolic modulation have been proposed to make T cells more responsive to antigen boosting by vaccination. Thus, a sequential or combined administration of different compounds with different complementary effects on protective immune responses may be necessary to counteract the number of inhibitory mechanisms that are known to be simultaneously operative in chronic HBV infection. This may represent a problem for in vivo application because safety is the first requirement for novel HBV therapies, especially in consideration of the optimal tolerability of available treatments. In addition, to what extent dysfunctional T and B cells which have been exposed for decades to different inhibitory mechanisms can be corrected is still an open issue and an insufficient cell functional reconstitution may represent a major limit to the curative potential of immune modulatory therapies. In this context, adoptive transfer of genetically engineered CD8 T cells produced in vitro either to recognize HLA/peptide complexes in an HLA restricted manner, or HBV antigens in a HLA independent fashion on infected liver cells, can allow to bypass the hurdle represented by functional T cell reconstitution. These treatments are however practically very difficult to apply in clinics; and moreover, adoptively transferred T cells may undergo the same suppressive effects triggered by the inflamed intrahepatic environment, which contribute to inactivate autologous HBV-specific CD8 cells (such as inhibition by PD-1/PD-L1 interaction), and may be associated with safety problems related to the size of cytotoxic T cell infusion. A possible solution may be the silencing of the key intracellular co-inhibitory pathways to relieve intrahepatic suppression and the amplification of the non-cytolytic potential of engineered CD8 T cells, which would make an HBV cure more effective but less dangerous for the infected liver. Thus, additional work is needed to find suitable solutions to limit the risk, improve the efficacy and simplify the clinical application of adoptive transfer interventions on the one hand, and to characterize better the features of T cell exhaustion on the other in the perspective of identifying more optimal strategies to reconstitute fully protective antiviral T and B cell functions.

## Figures and Tables

**Table 1:** Therapeutic Hepatitis B vaccines in clinical trials.

Vaccine Name	Vaccine Composition	Antiviral Treatment	Estimated Enrollment	Phase	Trial Registration	Findings Available	References
**TG1050**	Adeno vector encoding core, polymerase, envelope fusion protein	Add-on therapy to Tenofovir or Entecavir (>2 years)	48	Ib	NCT02428400	Completed; results not reported	
**GS-4774**	Heat-inactivated yeast containing S, core, X proteins	Add-on therapy to NUCs (>1 years)	178	II	NCT01943799	Completed; no significant HBsAg reduction	[77]
		Combined with Tenofovir	195	II	NCT02174276	Completed; no significant HBsAg reduction	[78]
**Theravax (DV-601)**	S, core proteins, Iscomatrix adjuvant	Combined with Entecavir	14	Ib	NCT01023230	Completed; anti-viral response observed	[79]
**ABX203**	S, core proteins,	Add-on therapy to NUCs	261	IIb/III	NCT02249988	Recruitment completed; results not reported	
		Vaccine versus Peg-IFN	160	III	NCT01374308	Recruitment completed; superior reduction of the viral load in Vaccine group	[80]
**Yeast-derived Immune Complexes, YIC**	HBsAg-hepatitis B immunoglobulin (HBIG); Alum as adjuvant	Untreated CHB patients	450	III		Recruitment completed; no difference between YIC group and placebo group in obtaining antiviral response	[81]
	HBsAg-hepatitis B immunoglobulin (HBIG); YIC versus alum alone	Combined with Adefovir	44	Pilot clinical study		Recruitment completed; anti-viral response observed in YIC group (rate of HBeAg seroconversion)	[82]
**HepTcell^TM^**	Peptides + IC31^®^ adjuvant	Add-on therapy to NUCs	60	I	NCT02496897	Recruitment completed; results not reported	
**ePA-44**	Multi-Peptides (HBV + tetanus toxoid)	Combined with Entecavir	378	II	NCT01326546	Recruitment status unknown; results not reported	
**INO-1800**	DNA plasmids encoding S and core	Add-on therapy to NUCs	90	I	NCT02431312	Recruitment completed; results not reported	
**HB-110**	DNA plasmids encoding HBs, PreS1, HBc, HBpol	Combined with Adefovir	27	I	NCT00513968	Recruitment completed; no significant rate of HBeAg seroconversion	[83]
		Combined with Entecavir	9	I	NCT01641536	Recruitment completed; results not reported	
**HB02 VAC-ADN**	DNA vaccine encoding preS/S	Add-on therapy to NUCs	70	I/II	NCT00536627	Recruitment completed; no change in relapse rate or decrease of virological breakthrough after therapy discontinuation	[84,85]
**pSG2.HBs/MVA.HBs**	DNA vaccine encoding HBsAg + modified vaccinia virus Ankara (MVA)	Alone or combined with Lamivudine	77	IIa	ISRCTN ISRCTN67270384	Recruitment completed; no antiviral response observed	[86]
**CVI-HBV-002**	DNA vaccine encoding S	Add-on therapy to NUCs	36	I/II	NCT02693652	Recruitment status unknown; results not reported	
**HPDCs-T immune therapy**	HBsAg activated dendritic cells	Combined with Peg-IFN or NUCs	450	I/II	NCT01935635	Recruitment status unknown; results not reported	
